# Identification and characterization of novel elastin gene mutations in eleven families with supravalvular aortic stenosis

**DOI:** 10.3389/fgene.2022.1059640

**Published:** 2022-11-28

**Authors:** Jianrong Zhou, Yueheng Wu, Xiaoli Xu, Yong Zhang, Xiong Zhang, Haisheng Chen, Jian Zhuang, Jimei Chen, Yun Teng

**Affiliations:** ^1^ Department of Cardiovascular Surgery, Guangdong Cardiovascular Institute, Guangdong Provincial People’s Hospital, Guangdong Academy of Medical Sciences, Guangzhou, China; ^2^ Department of Cardiovascular Surgery of Guangzhou First People’s Hospital, Guangzhou, China; ^3^ Guangdong Provincial Key Laboratory of South China Structural Heart Disease, Guangdong Provincial People’s Hospital, Guangdong Academy of Medical Sciences, Guangzhou, China; ^4^ State Key Laboratory of Medical Genomics, Key Laboratory for Endocrine and Metabolic Diseases of Ministry of Health, National Clinical Research Center for Metabolic Diseases, Rui-Jin Hospital, Shanghai Jiao Tong University School of Medicine, Shanghai, China

**Keywords:** ELN, supravalvular aortic stenosis, whole-exome sequencing, gene mutation, premature stop codons

## Abstract

**Background:** Supravalvular aortic stenosis (SVAS) is a rare congenital heart disease affecting approximately 1 in 25,000 live births. In some patients it is accompanied by pulmonary artery stenosis, particularly of pulmonary artery branches. Chronic stenosis can lead to cardiac hypertrophy and even circulatory failure. Familial autosomal dominant SVAS is frequently associated with elastin (ELN) gene mutations, whereas Williams-Beuren syndrome is a complex developmental disorder caused by heterozygous microdeletions of 26–28 genes at 7q11.23, including ELN.

**Methods:** Whole-exome sequencing was performed in 42 individuals from 11 Chinese families with SVAS to identify the pathogenic gene mutations involved. Aortic tissue was obtained for histological analyses, and quantitative reverse-transcription-PCR and western blotting were used to verify the expression of elastin molecules.

**Results:** Five point mutations and six frameshift mutations in the ELN gene were detected in the peripheral blood of all investigated families. Nine were nonsense mutations that result in premature stop codons, and the other two were missense mutations. All variants were heterozygous. Nine of the variants were novel, and have not been included in databases or previously reported. One mutation occurred in individuals from two different families. Reduced elastin protein expression was evident in patients’ aortic tissue.

**Conclusions:** The novel mutations of ELN were found to be pathogenic, which confirmed by reduced elastin expression and leads to SVAS. Thus, detailed cardiac testing and genetic counseling are warranted for patients and asymptomatic individuals with these mutations.

## Introduction

Supravalvular aortic stenosis (SVAS; Online Mendelian Inheritance in Man [OMIM] #185500) is a congenital heart disease with an incidence of approximately 1 in 25,000 live births (Ewart et al., 1993). Peripheral pulmonary stenosis (PPS) is known to occasionally coexist with SVAS, and induces increased resistance to blood flow and causes elevated ventricular pressure and hypertrophy resulting in heart failure (Collins et al., 2010).

SVAS is the main feature of elastin arteriopathy, and is related to haploinsufficiency of the elastin (*ELN*) gene located at 7q11.23 that encodes the elastin protein (Denie and Verheugt, 1958). The syndromic form, Williams-Beuren syndrome (WBS; OMIM #194050), accounts for approximately 30%–50% of SVAS patients. It is a complex genetic disorder caused by heterozygous microdeletions on chromosome 7 at 7q11.23, characterized by intellectual disability, hypercalcemia, impaired social interactions, facial dysmorphism, and SVAS (Collins, 2018; Duque Lasio and Kozel, 2018; Williams syndrome, 2021). Autosomal dominant non-syndromic “familial SVAS” accounts for 20% of SVAS patients. The *ELN* gene which encodes elastin is causatively involved in non-syndromic familial SVAS. Hemizygosity for elastin causes SVAS, and sometimes peripheral artery stenosis and hernias, but not the other features of WBS (Metcalfe et al., 2000; Hayano et al., 2019). SVAS can also occur sporadically with unknown etiology.

To date more than 100 pathogenic or suspected pathogenic mutations of the *ELN* gene have been described in the literature, the ClinVar database (https://www.ncbi.nlm.nih.gov/clinvar) and the human gene mutation database (http://www.hgmd.cf.ac.uk) (Duque Lasio and Kozel, 2018). *ELN* mutations mainly include missense mutations affecting methionine, nonsense mutations, and frameshift mutations resulting from insertions and deletions. Previous studies indicate that point mutations can lead to premature stop codons (PTCs), resulting in functional elastin haploinsufficiency through a nonsense-mediated mRNA decay mechanism (Micale et al., 2010; Hayano et al., 2019). It remains unclear whether *ELN* can explain the remaining cases of SVAS, or whether there are unidentified causative genes. The molecular mechanism of the disease requires further study.

The current study included 42 patients with SVAS from 11 families. *ELN* mutations were assessed *via* whole-exome sequencing (WES) to elucidate the genetic background of SVAS. Aortic tissues were collected from some patients after surgery to further define the molecular pathology of familial SVAS.

## Materials and methods

### Ethical compliance

The research plan was approved by the Research Ethics Committee of Guangdong Provincial People’s Hospital (no. GDREC2019587H(R1)). The study protocol conformed to the ethical guidelines of the 1975 Declaration of Helsinki as reflected by *a priori* approval from the institution’s human research committee. Written informed consent was obtained from all patients or their parents.

### Patients and samples

Eleven Chinese families with SVAS were included in the study. None of the probands had growth retardation, abnormal facial features, or chromosome abnormalities as determined by chromosomal microarray analysis (CMA). SVAS and PPS were diagnosed *via* echocardiography when the pressure gradient at the stenosis exceeded 10 mmHg (Cha et al., 2019). After obtaining informed consent from the patient or parents, approximately 2.0 ml of peripheral venous blood was collected from the proband and family members. Genomic DNA was extracted using the QIAamp DNA Mini Kit (QIAGEN GmbH, Germany) in accordance with the manufacturer’s instructions. The probands from families A, B, and I with severe SVAS were underwent cardiac surgery in our center, then the stenotic ascending aorta tissues were obtained from the patients after surgery. Normal control tissue was taken from the ascending aortas of three sex and age-matched donors undergoing heart transplantation.

### Whole-exome sequencing analysis

Agilent’s liquid chip capture system (Agilent Technologies, Santa Clara, CA, Uniteds States) was used to enrich the whole exon region in each sample, then high-throughput and high-depth sequencing was performed on the Illumina platform (San Diego, CA, Uniteds States). The Agilent SureSelect Human All Exon V6 (Agilent Technologies) was used to build and capture the database. Only the reagents and consumables recommended in the manual were used, and the process was conducted in accordance with the latest optimized experimental process. Sequencing data were compared to the reference gene (grch37/hg19) *via* BWA software (version 0.7.8-r455) (Li and Durbin, 2009). Based on the comparison results, SMAtools (version 1.0) (Li et al., 2009) was used to identify single-nucleotide polymorphism sites and InDels; the results were filtered using international commonly used filtering standards, and copy number variation was detected by CoNIFER software (version 0.3) (http://sv.gersteinlab.org/cnvnator/). Annovar (Wang et al., 2010) was used to annotate genetic variation.

### Variant confirmation using sanger sequencing

Sanger sequencing was performed on a subset of patients to validate mutations expected to be pathogenic. The target region of *ELN* was sequenced, and sequence data were obtained *via* the BigDye^®^ Terminator Version 3.1 Cycle Sequencing Kit (Thermo Fisher Scientific) and a 3730xl automatic sequencer (Gene Tools, Uniteds States). The results were analyzed *via* BioEdit software (version 7.2.5). Primer sequences are listed in Supplementary Table S1.

### Microscopic examination

Surgically resected aortic tissue was fixed in 4% paraformaldehyde for 24 h then embedded in paraffin. Trimmed wax blocks were placed on a paraffin microtome (Thermo Fisher Scientific, Waltham, Uniteds States, HM325) by the pathology laboratory of Biossci (Hubei, China) Biotechnologies Company Ltd., and 4 μm-thick pieces were continuously cut. Sections were stained with elastin-van Gieson and immunofluorescence. Anti-elastin antibody (Abcam, ab213720, 1:1000) was used as primary antibody. Images were obtained using the HAMAMATSU imaging system (C13220-0, Japan). Measurements were performed three times.

### Quantitative reverse transcription PCR

Total RNA was extracted from aortic tissue *via* the TRIzol method, and quantitative real-time reverse transcription PCRs (qRT-PCRs) reactions for the *ELN* gene were performed using the premix Pro Taq HS qPCR II Kit (Accurate Biosciences) in accordance with the manufacturer’s instructions. Gene expression of interest was quantified using the comparative cycle threshold (CT). Relative amounts of gene mRNA were determined by subtracting the CT value of the gene of interest from the CT value of the housekeeping gene GAPDH (∆ CT) (Livak and Schmittgen, 2001). Primers were designed and synthesized by Shanghai Shenggong *via* Primer 5.0 software, with the housekeeping gene GAPDH as the internal reference. Primer sequences are listed in Supplementary Table S2.

### Western blotting

Arterial tissue samples were rapidly frozen in liquid nitrogen within 5 min of acquisition. Tissue blocks were washed 2–3 times with pre-chilled PBS to remove blood contamination, sheared into small pieces and placed in homogenization tubes with two 3–mm homogenization beads and ten times the tissue volume of lysis solution. Protease inhibitors were added within the first few minutes. After homogenization the completed homogenate was removed and placed in lysis buffer on ice for 30 min, and shocked every 5 min to ensure complete tissue lysis. The lysate was then centrifuged at 12,000 rpm at 4°C for 10 min, and the supernatant was collected, that is, the total protein solution. The protein solution was added to SDS buffer at a ratio of 4:1 and denatured in a boiling water bath for 15 min. Equal amounts of protein for each sample were separated by SDS-PAGE then electrophoretically transferred to PVDF membranes. The membranes were then incubated with primary antibodies in accordance with the antibody manufacturer’s instructions, with the primary antibody used coinciding with the aforementioned immunofluorescence. This was followed by a horseradish peroxidase-conjugated secondary antibody (Servicebio). GAPDH was used as a control for protein loading.

### Statistical analysis

All data were expressed as mean ± the standard error of the mean. Comparisons between groups were performed using student’s *t*-test with the least significant difference test. *P* values were two tailed, and < 0.05 was considered statistically significant. All data were analyzed using SPSS 22.0 software (IBM Corp., Armonk, NY).

## Results

### Study population characteristics

**FIGURE 1:**
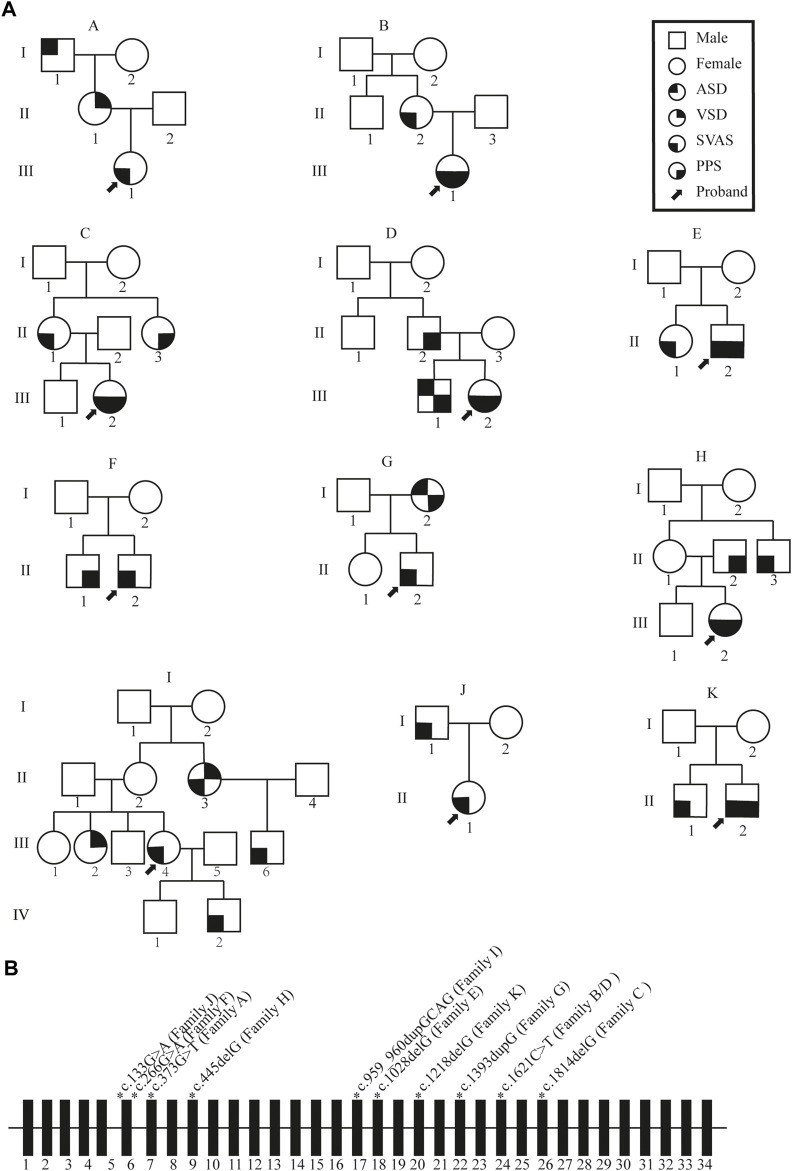
Schematic representation of the familial mutation. **(A)** Pedigree diagram of familial supravalvular aortic stenosis. Arrows indicate probands. I, II, III and Ⅳ correspond to first, second, third and fourth generation, respectively. SVAS, supravalvular aortic stenosis; PPS, peripheral pulmonary stenosis; VSD, ventricular septal defect; ASD, atrial septal defect. **(B)** Schematic representation of the ELN gene mutations. Rectangles represent exons; thin horizontal line represents introns. The schematic position of the identified mutations is indicated with a star.

The cohort included 42 individuals from 11 SVAS pedigrees (Figure 1A). Retrospective analyses of medical history data from each proband and their family members were performed. Clinical manifestations were mainly associated with SVAS, and some patients also had PPS. All probands had normal neuro-intellectual and physical development. Three probands underwent surgical correction of SVAS at our center.

### Genotype characteristics

**FIGURE 2:**
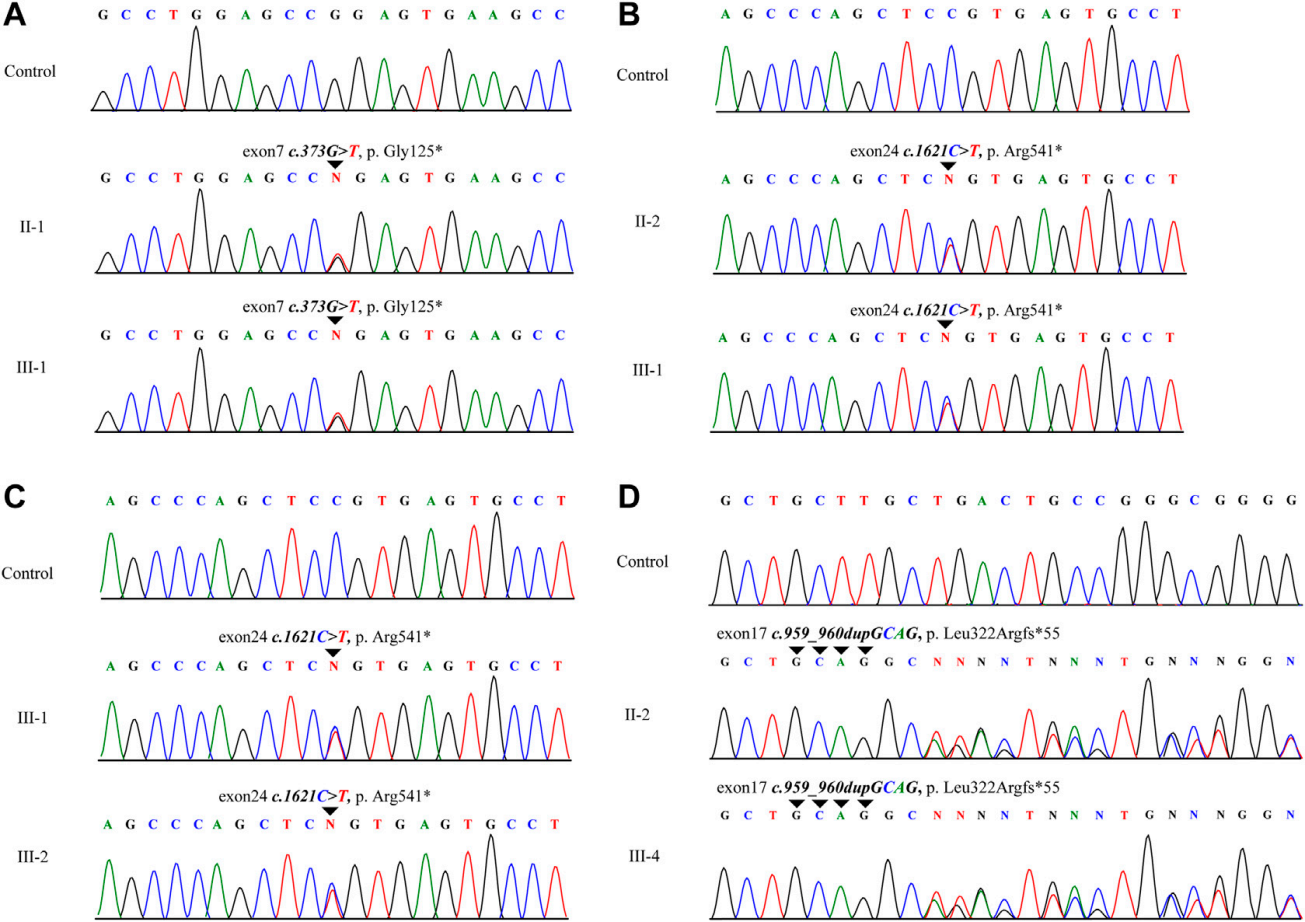
Schematic of a subset of the ELN mutations identified in this study. The schematic positions of identified mutations are represented by triangles. **(A–D)** represent families A, B, D and I. II and III correspond to second and third generation, respectively.

Whole-exon sequencing was performed on all individuals participating in the study. No associated pathogenic copy number variation was observed. All families had variants of the *ELN* gene, which were determined to be pathogenic according to the American College of Medical Genetics criteria (ACMG) (Miller et al., 2021). All variants were heterozygous, and nine of the variants were novel in that not included in any databases or previously described. Two different families had the same mutation. Details of the *ELN* gene mutations and associated patient phenotypes are presented in Figure 1B; Table 1. To validate the results of WES, Sanger sequencing was performed on available family members (Figure 2).

**TABLE 1:** Phenotype and spectrum of *ELN* gene mutations.

Family	Subject	Nucleotide change	dbSNP	Location	Amino-acid change	Clinical phenotypes
A	I-1	c.373G>T	Novel	exon7	p. Gly125^*^	ASD
	I-2	—		—	—	—
	II-1	c.373G>T	Novel	exon7	p. Gly125^*^	PPS
	II-2	—		—	—	—
	III-1	c.373G>T	Novel	exon7	p. Gly125^*^	SVAS
B	II-2	c.1621C>T	rs137854453	exon24	p. Arg541^*^	SVAS
	II-3	—		—	—	—
	III-1	c.1621C>T	rs137854453	exon24	p. Arg541^*^	SVAS, PPS
C	I-1	c.1814delG	Novel	exon26	p. Gly605Valfs^*^70	Nonpenetrant
	I-2	—		—	—	—
	II-1	c.1814delG	Novel	exon26	p. Gly605Valfs^*^70	SVAS, CAE
	II-3	c.1814delG	Novel	exon26	p. Gly605Valfs^*^70	PPS, IH
	III-1	—		—	—	—
	III-2	c.1814delG	Novel	exon26	p. Gly605Valfs^*^70	SVAS, PPS
D	II-2	c.1621C>T	rs137854453	exon24	p. Arg541^*^	PPS
	II-3	—		—	—	—
	III-1	c.1621C>T	rs137854453	exon24	p. Arg541^*^	ASD, PPS
	III-2	c.1621C>T	rs137854453	exon24	p. Arg541^*^	SVAS, PPS
E	I-1	c.1028delG	Novel	exon18	p. Gly343Valfs^*^121	Nonpenetrant
	I-2	c.1028delG	Novel	exon18	p. Gly343Valfs^*^121	SVAS
	II-2	c.1028delG	Novel	exon18	p. Gly343Valfs^*^121	SVAS, PPS
F	II-1	c.266G>A	Novel	exon6	p. Gly89Glu	PPS
	II-2	c.266G>A	Novel	exon6	p. Gly89Glu	SVAS
G	I-1	—	—	—	—	—
	I-2	c.1393dupG	Novel	exon22	p. Ala465Glyfs^*^127	ASD, PPS
	II-2	c.1393dupG	Novel	exon22	p. Ala465Glyfs^*^127	SVAS
H	I-2	c.445delG	Novel	exon9	p. Val149Tyrfs^*^34	SVAS
	II-2	c.445delG	Novel	exon9	p. Val149Tyrfs^*^34	PPS
	II-3	c.445delG	Novel	exon9	p. Val149Tyrfs^*^34	SVAS
	III-1	—		—	—	—
	III-2	c.445delG	Novel	exon9	p. Val149Tyrfs^*^34	SVAS, PPS
I	II-2	c.959_960dupGCAG	Novel	exon17	p. Leu322Argfs^*^55	Nonpenetrant
	II-3	c.959_960dupGCAG	Novel	exon17	p. Leu322Argfs^*^55	SVAS, VSD
	II-4	—		—	—	—
	III-2	c.959_960dupGCAG	Novel	exon17	p. Leu322Argfs^*^55	PPS
	III-4	c.959_960dupGCAG	Novel	exon17	p. Leu322Argfs^*^55	SVAS, CAE
	Ⅳ-2	c.959_960dupGCAG	Novel	exon17	p. Leu322Argfs^*^55	SVAS
J	I-1	c.133G>A	Novel	exon6	p. Gly45Arg	SVAS
	II-1	c.133G>A	Novel	exon6	p. Gly45Arg	SVAS
K	I-2	c.1218delG	Novel	exon20	p. Phe407 Leufs^*^57	SVAS
	II-1	c.1218delG	Novel	exon20	p. Phe407 Leufs^*^57	SVAS
	II-2	c.1218delG	Novel	exon20	p. Phe407 Leufs^*^57	SVAS, PPS

Abbreviations: ASD, atrial septal defect; CAE, coronary artery ectasia; IH, inguinal hernia; PPS, peripheral pulmonary stenosis; SVAS, supravalvular aortic stenosis; VSD, ventricular septal defect. I, II, III and Ⅳ correspond to first, second, third and fourth generation, respectively. All mutations were heterozygous.

### Reduced expression of elastin in the aorta of *ELN*-mut patients

**FIGURE 3:**
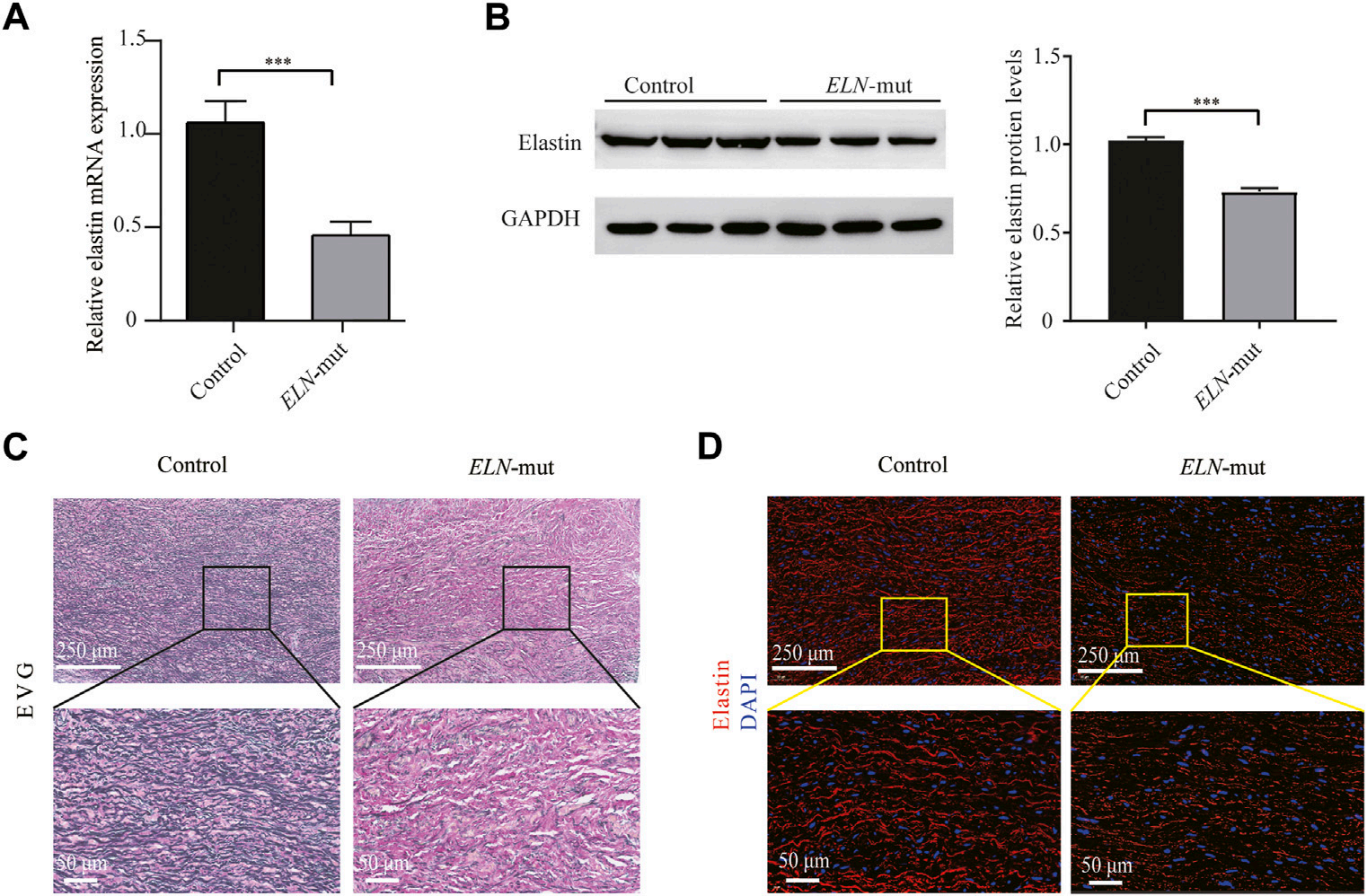
Expression analysis of *ELN* gene in aortic tissues. **(A)** Expression levels of *ELN* mRNA in the *ELN*-mut patients’ aortic tissue. **(B)** Elastin levels in aortic tissue of patients. **(C,D)** EVG stain and immunofluorescence of arterial tissues. Tissue samples were obtained from three normal subjects and three probands (from families A, B, and I) with severe SVAS, and statistical methods were performed using student’s *t*-test. ***, *p*< 0.01.

*ELN* mRNA transcription levels in aortic tissues were reduction by approximately half compared with normal tissues (Figure 3A). Elastin protein expression levels were also reduced as evidenced by western blotting (Figure 3B). EVG staining of arterial tissue revealed that the elastin content of the tunica media of arterial tissue was significantly lower in SVAS patient aortic tissue than in aortic tissue from healthy controls (Figure 3C). There was a lower percentage of elastin-positive area in the aortic walls in *ELN*-mut patients. Aortic tissues from *ELN*-mut patients lacked intact elastin lamellae, and contained elastic fibers that were disorganized and fragmented compared with controls (Figure 3D).

## Discussion

Mutation or microdeletion within the *ELN* gene can lead to SVAS (Duque Lasio and Kozel, 2018). In an attempt to elucidate the molecular pathology of SVAS we performed mutation screening of the *ELN* gene. Pathogenic mutations of the *ELN* gene were found in 11 autosomal dominant SVAS families, and nine of them were novel mutations that had not been reported and were not included in any database. Two different families had the same mutation. Among the 11 SVAS families, nine had nonsense mutations and 2 had missense mutations. Analysis of aortic tissue revealed reduced elastin expression. These results suggest that reduced elastin expression caused by heterozygous pathogenic mutations in the *ELN* gene is the major genetic cause in hitherto unexplained cases of familial SVAS in China.

Elastin is encoded by the *ELN* gene and is expressed in a variety of tissues and organs including large artery smooth muscle cells, and it contributes to tissue elasticity (Yeo et al., 2016). Although the molecular mechanism of SVAS has not been fully elucidated, it is mainly related to insufficient secretion of elastin by aortic smooth muscle cells caused by *ELN* gene mutation or deletion (Hayano et al., 2019; Min et al., 2020; Dave et al., 2022). Consistent with previous studies (Micale et al., 2010), in the current cohort of patients the disease was mainly caused by nonsense mutations in the *ELN* gene leading to PTCs. Analysis of aortic mRNA expression in the patients with PTC mutations showed that haploinsufficiency of *ELN* expression caused SVAS and nonsense mutation-mediated mRNA degradation. However, no differences in ELN expression levels in aortic tissue between patients with different mutations were found in this study. This may be related to the fact that the sample size of our study was not very large, which remains to be further explored in future studies.

The same mutation was found in two different families (c.1621C>T in families B and D), and this mutation has also been reported in ClinVar. Therefore, we hypothesized that this locus may be a hotspot for mutation of SVAS patients.

The main clinical phenotypes of patients with *ELN* mutations are arterial stenosis, especially SVAS and pulmonary artery stenosis (Keating, 1994; Park et al., 2006). These symptoms are similar to those in patients with WBS (Eronen et al., 2002; Delio et al., 2013) thus the two groups of patients are sometimes difficult to distinguish based on clinical findings alone. In the present study some patients were initially suspected of having WBS, but no abnormalities were found after Fluorescence *in situ* hybridization (Ramirez-Velazco et al., 2019), multiplex ligation-dependent probe amplification (Honjo et al., 2015), and chromosomal microarray analysis (Kuo et al., 2019) targeting probes containing the WBS chromosomal region. Therefore, in such patients the use of methods with higher sequencing depth is recommended, such as whole-exon sequencing and whole-genome sequencing, to increase the gene mutation detection rate.

In most individuals in the present study there was a consistent separation of symptoms and mutations, and most mutations resulted in PTCs. However, there were also individuals in the same family with *ELN* mutations who did not suffer from cardiovascular malformations or present with other types of defects. Moreover, no other related genes mutation was found according to the whole exome sequencing data that may explain this clinical manifestation variation. These cases are consistent with previous studies (Metcalfe et al., 2000; Jakob et al., 2011; Jelsig et al., 2017) in which SVAS families had highly variable cardiovascular phenotypes ranging from asymptomatic to multiarterial severe stenosis. Such differences may be related to epigenetics (Parrish et al., 2020; Procknow and Kozel, 2022), or some individuals may initially have undetected arterial stenosis then undergo self-healing, suggesting that pulmonary artery stenosis caused by elastin depletion has a high probability of undergoing gradual improvement as reported by Collins et al. (2010). Taken together, findings to date indicate that cardiac screening and genetic counseling should be administered to relatives of SVAS patients, as unaffected carriers may give birth to severely affected individuals.

Consistent with previous findings (Micale et al., 2010) PTC mutations were associated with *ELN* mRNA substrate deficiency, resulting in reduced elastin expression, which may be caused by nonsense-mediated attenuation of PTC mutations. Interestingly, we also identified two families with *ELN* missense mutations, which are not common in previous reports. We suspect that this may be related to normal splicing affecting *ELN* gene transcription, resulting in truncation of the protein (Zhang et al., 1999; Kozel et al., 2003; Ott et al., 2011).

## Conclusion

The current study indicates that point mutations within the *ELN* gene are an important genetic cause of familial SVAS in China. The use of whole-exome sequencing or whole-genome sequencing gene testing technology is therefore recommended for genetic diagnosis in such patients, to improve the mutation detection rate. The study also illustrates the importance of screening for *ELN* gene mutations in patients with arterial stenosis, especially SVAS and pulmonary stenosis, in order to identify the genetic etiology involved. Notably, our findings revealed that insufficient elastin expression is the main cause of these vascular lesions. Further investigation is required to identify the specific molecular mechanism involved however, which may in turn contribute to the identification of potentially therapeutic drug targets.

## Data Availability

The original contributions presented in the study are included in the article/Supplementary Material, further inquiries can be directed to the corresponding authors.
