# Supplementary material for: Identification and characterization of novel elastin gene mutations in eleven families with supravalvular aortic stenosis
Source: Front Genet. 2022 Nov 28;13:1059640. doi: 10.3389/fgene.2022.1059640 (PMC9742416; doi:10.3389/fgene.2022.1059640)
Supplement: Supplementary file 1 [file Table1.DOCX]

**Supplementary Table 1. PCR Primers Used to Verify *ELN* Gene Mutations**

| Family | Target | Forward primer | Reverse primer |
| --- | --- | --- | --- |
| A | c.373G>T | 5'-TACGCAATGCCTCACCTGTC- 3' | 5'-GACTCGCCTCTTTGAGCCTC-3' |
| B | c.1621C>T | 5' TTGCAGGTGAGTTTCATGAGTC 3' | 5' TGCAGACAGAGGGCAGTGGT 3' |
| D | c.1621C>T | 5' TTGCAGGTGAGTTTCATGAGTC 3' | 5' TGCAGACAGAGGGCAGTGGT 3' |
| I | c.959_960dupGCAG | 5' TTGGCTTCTCTTGGCTTCTTG 3' | 5' GGGCAACCTCTCAAATACACTG 3' |
