# Supplementary material for: Identification and characterization of novel elastin gene mutations in eleven families with supravalvular aortic stenosis
Source: Front Genet. 2022 Nov 28;13:1059640. doi: 10.3389/fgene.2022.1059640 (PMC9742416; doi:10.3389/fgene.2022.1059640)
Supplement: Supplementary file 2 [file Table2.DOCX]

**Supplementary Table 2. PCR primers for detection of *ELN* gene transcript levels**

| Primer name | Primer sequences | Length |
| --- | --- | --- |
| ELN-human-F | GCAGGAGTTAAGCCCAAGG | 19 |
| ELN-human-R | TGTAGGGCAGTCCATAGCCA | 20 |
| GAPDH-human-F | ACAACTTTGGTATCGTGGAAGG | 22 |
| GAPDH-human-R | GCCATCACGCCACAGTTTC | 19 |
